# Graphene transistors for real-time monitoring molecular self-assembly dynamics

**DOI:** 10.1038/s41467-020-18604-4

**Published:** 2020-09-18

**Authors:** Marco Gobbi, Agostino Galanti, Marc-Antoine Stoeckel, Bjorn Zyska, Sara Bonacchi, Stefan Hecht, Paolo Samorì

**Affiliations:** 1 IKERBASQUE, Basque Foundation for Science, 48013 Bilbao, Basque Country Spain; 2 CIC nanoGUNE BRTA, 20018 Donostia-San Sebastian, Basque Country Spain; 3 Centro de Física de Materiales CFM-MPC (CSIC-UPV/EHU), 20018 Donostia-San Sebastian, Basque Country Spain; 4 University of Strasbourg, CNRS, ISIS UMR 7006, 8 allée Gaspard Monge, 67000 Strasbourg, France; 5 Department of Chemistry and IRIS Adlershof, Humboldt-Universität zu Berlin, 12489 Berlin, Germany; 6 Department of Chemical Sciences, University of Padua, Via Francesco Marzolo 1, 35131 Padova, Italy; 7 DWI—Leibniz Institute for Interactive Materials, Forckenbeckstr. 50, 52074 Aachen, Germany; 8 Institute of Technical and Macromolecular Chemistry, RWTH Aachen University, Worringer Weg 2, 52074 Aachen, Germany

**Keywords:** Self-assembly, Electronic properties and devices, Two-dimensional materials

## Abstract

Mastering the dynamics of molecular assembly on surfaces enables the engineering of predictable structural motifs to bestow programmable properties upon target substrates. Yet, monitoring self-assembly in real time on technologically relevant interfaces between a substrate and a solution is challenging, due to experimental complexity of disentangling interfacial from bulk phenomena. Here, we show that graphene devices can be used as highly sensitive detectors to read out the dynamics of molecular self-assembly at the solid/liquid interface in-situ. Irradiation of a photochromic molecule is used to trigger the formation of a metastable self-assembled adlayer on graphene and the dynamics of this process are monitored by tracking the current in the device over time. In perspective, the electrical readout in graphene devices is a diagnostic and highly sensitive means to resolve molecular ensemble dynamics occurring down to the nanosecond time scale, thereby providing a practical and powerful tool to investigate molecular self-organization in 2D.

## Abstract (web summary)

Molecular self-assembly provides the desired functions to substrates, but investigation and control of its dynamics is challenging for the large area over which it must be detected. Here the authors report the use of graphene field effect devices to monitor with sub-second time resolution the photoinduced supramolecular assembly of a spiropyran derivative on graphene, covering an area of 100 × 100 μm^2^.

## Introduction

Molecular self-assembly on surfaces generates highly ordered 2D structures^1–3^, which are capable to impart desired functions to a substrate^4,5^. As the imparted functions depend on the arrangement on the molecular scale, scanning probe microscopy techniques have been widely employed to map in the direct space the architectural motifs obtained through self-assembly^6–12^. The latter, which is ruled by a complex interplay between intramolecular, intermolecular, and interfacial interactions^13^ is not completely understood^14,15^.

Unraveling the dynamics of self-assembly^16^ could provide higher control over key parameters governing the mechanism of molecular self-organization in 2D, thereby permitting to further engineer molecular functionalization^7,17^. Scanning tunneling microscopy (STM) imaging enabled to monitor with sub-nanometer spatial resolution the kinetics of nucleation and rearrangements taking place in supramolecular adlayers at solid/liquid interfaces^18–21^, including light-responsive assemblies composed of photochromic molecules^22–26^. However, the information provided by STM is confined at a length scale of a few tens of square nanometers, thus not suitable to describe population dynamics of self-assembly on macroscopic distances, which involves billions of molecules. Moreover, the highest temporal resolution of STM is limited by the ability to record a few tens of frames per second^16,27^, yielding a temporal resolution of 10–100 ms, or slower (1–10 s) when it comes to visualizing molecular assemblies^16^.

For this reason, the use of a solely electrical read out to track the ensemble dynamics of molecular self-assembly would be a highly desirable tool to attain ultrafast response and insight into the phenomena governing self-organization in 2D. While electronic devices were employed to monitor in real time single-molecule reactions^28^ and DNA hybridization^29^, the dynamics of a complex ensemble process such as the on-surface self-assembly has not been read out by means of an electronic device.

Here, we demonstrate that graphene field-effect devices represent a powerful tool to monitor electrically in an easy and controllable way the complex dynamics of on-surface self-assembly of photochromic molecules. As a proof of principle, we employ a molecule that upon irradiation generates a metastable isomer capable of forming a supramolecular assembly at the graphene/solution interface, thereby introducing a light-controlled field effect on graphene analogous to that of an external gate terminal. Therefore, by measuring the temporal evolution of the electrical current flowing through graphene, we are capable to track the dynamics of formation and desorption of the metastable self-assembled monolayer. Importantly, we demonstrate that the ultrahigh surface sensitivity of graphene permits to disentangle the dynamics of self-assembly at the solid–liquid interface from those of ensemble processes taking place simultaneously in the supernatant solution, such as photoisomerization and thermal relaxation.

## Results

### Optical characterization of photoisomerization in solution

**Fig. 1:**
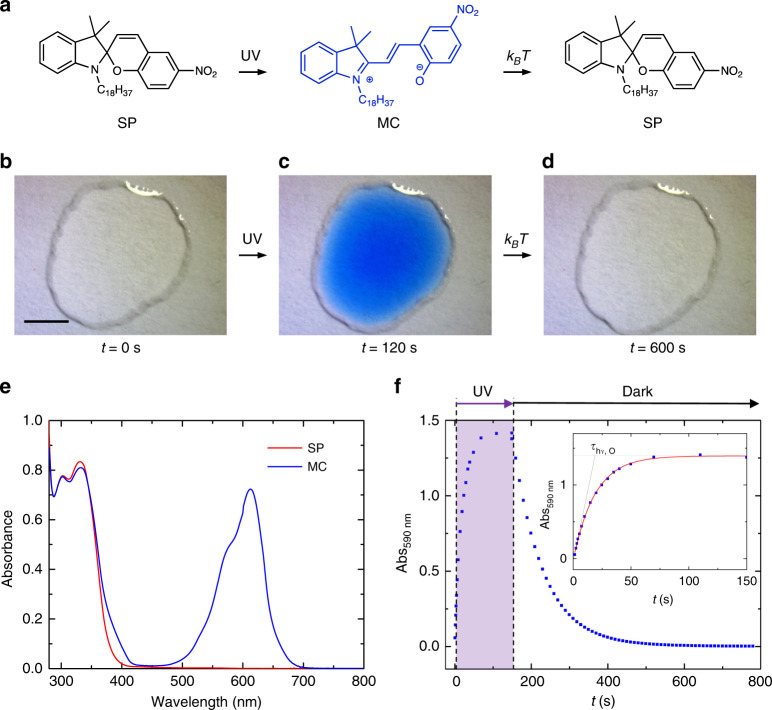
Optical characterization of the photoswitching dynamics. **a** Reversible photochemical and thermal interconversion of spiropyran (SP) derivative and its merocyanine (MC) isomer employed in this work. Optical micrographs of a 4 μL drop of SP solution in 1-phenyloctane (concentration *C*_0_ = 0.9 mM), highlighting the color change (**b**) before and (**c**) after photoisomerization, triggered by 120 s of 365 nm irradiation with ultraviolet light (UV) as well as (**d**) after thermal reconversion, achieved by keeping the drop 10 min in the dark (*k*_B_*T*) (scale bar: 2 mm). (**e**) Absorption spectra of a SP/MC solution in 1-phenyloctane (*C*_0_ = 0.09 mM) before (red trace) and after 6 s of 365 nm UV irradiation (blue trace). **f** Kinetics of the color change of an SP/MC solution in 1-phenyloctane (*C*_0_ = 1 mM, 1 mm optical path cell), as monitored by plotting the absorbance at 590 nm during 150 s of 365 nm UV irradiation (*P* = 0.5 mW cm^−2^ illustrated by the purple area) and afterwards in the dark. The inset shows the evolution of Abs_590nm_ during UV light irradiation (blue squares) and the applied monoexponential fit (red line). The corresponding optical time constant *τ*_hν,O_ is deduced form the fit, and it can graphically be depicted as the intersection between the saturation value in Abs_590nm_ (dashed line) and the linear fit at the initial stage of irradiation (solid line).

For this study, we employed the spiropyran (SP) derivative shown in Fig. 1a. The octadecyl side chain promotes self-assembly on graphene by forming crystalline lamellae^5^. Irradiation with ultraviolet (UV) light at 365 nm triggers the conversion to the ring-open zwitterionic merocyanine (MC) isomer, which is metastable and thermally reverts back to the ring-closed SP isomer^30^. The photoisomerization is accompanied by a drastic change in the physical properties of the molecule, with the MC isomer being characterized by a stronger molecular dipole moment.

The SP → MC photoisomerization and the MC → SP thermal back-switch are most conveniently visualized by a change of color, as the transparent SP solution progressively turns blue under UV irradiation^30^, as shown in Fig. 1b–d for a small drop of SP solution in 1-phenyloctane. Importantly, 1-phenyloctane is a high boiling point solvent, hence its evaporation can be neglected on the few-minutes time scale of the experiments described in this work.

Figure 1e shows that the typical structured band of MC chromophores centered at 600 nm appears after a few seconds of irradiation. Therefore, the kinetics of the SP → MC photoisomerization during UV irradiation and the MC → SP conversion at room temperature can be followed by monitoring the evolution of the absorbance at 590 nm (hereafter Abs_590nm_). During UV irradiation, Abs_590nm_ rises until a photostationary state is reached after 90 s of UV irradiation, and, subsequently, it decreases during thermal relaxation (Fig. 1f), in agreement with the typical behavior of SP-based photoswitches in apolar solvents^30,31^. In light of the SP → MC first order photochemical reaction, the photoisomerization kinetics can be fitted by simple exponential curves in the form Abs_590nm_ (*t*) = Abs_sat_ − *B* × exp (−*t*/*τ*_hν,O_), where *τ*_hν,O_ is the time constant which characterizes the dynamics of the photoisomerization (inset in Fig. 1f and Supplementary Fig. 1). For the SP → MC photoisomerization kinetics monitored in our experimental conditions (“Methods”), the reaction is characterized by a time constant *τ*_hν,O_ = 18 s. Importantly, *τ*_hν,O_ was found to be independent on the initial SP concentration (*C*_0_) at the same photon flux (see Supplementary Fig. 1 and Supplementary Table 1). Note that the optical measurements displayed in Fig. 1 characterize the molecular events taking place in the bulk solution, hence they do not provide insights into the events occurring at the interface between the solution and any solid support.

### Molecular self-assembly at the solid/liquid interface

**Fig. 2:**
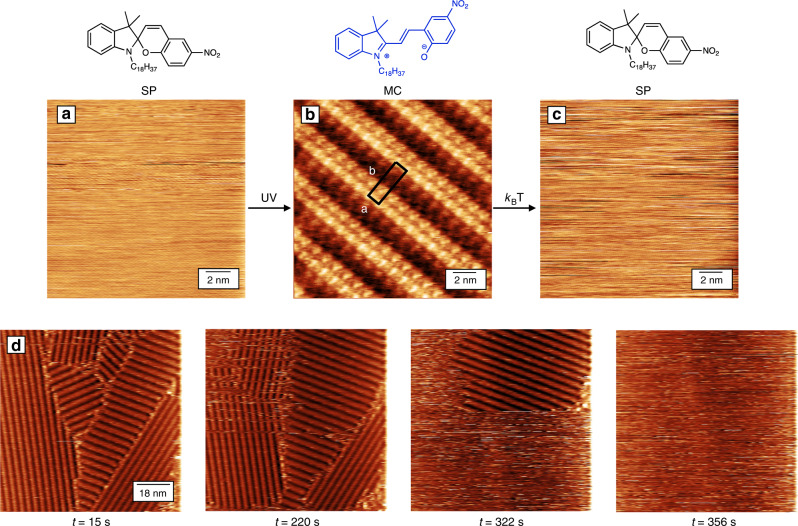
Scanning tunneling microscopy images of the SP/MC assemblies at the solid/liquid interface. **a** Ordered assemblies of the SP isomer could not be monitored by means of STM at the interface between highly oriented pyrolytic graphite (HOPG) and a 6 μL drop of SP/MC solution in 1-phenyloctane (*C*_0_ = 1.8 mM). **b** In situ 365 nm UV irradiation of the same drop (*P* = 1.7 mW cm^−2^) leads to SP → MC isomerization. Immediately after 60 s irradiation, a highly ordered MC assembly was visualized by STM. **c** 10 min after UV irradiation of the same droplet, the MC assembly could not be imaged by STM. **d** Large area, survey STM images consecutively acquired in the same spot showing the evolution of the MC disassembly. Different crystalline domains change size and shape in consecutive images, eventually leading to the disappearance of the assembly from the surface. At the bottom of each image, the time delay after switching off the UV light is displayed. The images were recorded with the STM tip immersed in a drop of SP/MC solution (*C*_0_ = 1.8 mM) in 1-phenyloctane, on HOPG. **a**–**c** Height images; tunneling current *I*_T_ = 60 pA, tunneling voltage *V*_T_ = 600 mV; **d** *I*_T_ = 20 pA, *V*_T_ = 600 mV.

In order to characterize interface events, we performed STM imaging at the interface between highly oriented pyrolytic graphite (HOPG) and a drop of SP solution in 1-phenyloctane. At the solid/liquid interface, we could not image any stable assembly for the SP isomer (Fig. 2a). This finding indicates that physisorbed SP molecules, which are nonplanar and chiral, do not form a firmly immobilized self-assembled structure at the solid/liquid interface due to their low affinity for the basal plane of HOPG^26^.

A very different scenario is encountered when the supernatant solution was irradiated with UV light to trigger the SP → MC isomerization. Immediately after irradiation, an ordered assembly could be imaged with sub-molecular resolution (Fig. 2b), displaying a lamellar assembly in which the long alkyl chains lie flat on the HOPG surface, as typically observed for alkyl chains on graphitic surfaces^2,25^. The unit cell of the assembly, as extracted from STM image analysis, is composed of two MC molecules and characterized by lattice parameters *a* = 1.1 ± 0.1 nm, and *b* = 3.9 ± 0.1 nm, forming an angle *α* = 90 ± 2° leading to an area *A* = 4.3 ± 0.2 nm^2^. Within the experimental error, the unit cell extracted from STM image analysis is the same as that previously measured at the solid–air interface for the same molecule, indicating that the assembly structure is similar in both cases^26^. A few minutes after UV irradiation, the assembly could not be any longer imaged by STM (Fig. 2c), since, due to the thermal MC → SP conversion in solution, the MC concentration decreases below the critical concentration necessary for assembly, thereby driving desorption of MC from the surface. By recording different STM images centered at the same location, we could follow how the self-assembled structure evolves and eventually disappears after ~6 min (Fig. 2d, see also Supplementary Fig. 2). Despite its high spatial resolution, STM only provides a rudimentary estimate for the dynamics of the self-assembly process in the limited region under investigation.

### Electrical detection of self-assembly dynamics

**Fig. 3:**
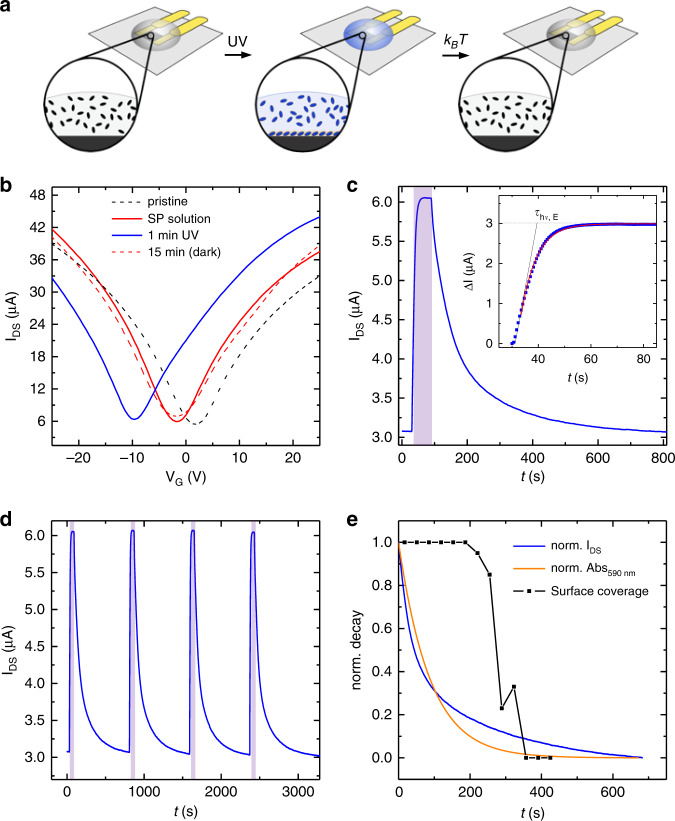
Electrical characterization of the self-assembly dynamics. **a** Schematic representation of our approach to monitor interfacial merocyanine (MC) self-assembly by the electrical current flowing in graphene. A droplet of spiropyran (SP) solution is casted onto a graphene device, and UV light is employed to trigger the SP → MC photoisomerization and consequently the formation of the MC self-assembled adlayer. After UV light irradiation, MC molecules thermally switch back to SP, leading to the dissolution of the MC assembly. The time evolution of the current flowing through the device allows monitoring the dynamics of formation and dissolution of the MC adlayer. **b** Drain current *I*_DS_ versus gate voltage *V*_G_ measured on a micromechanically exfoliated single layer graphene device before and after casting a drop of SP solution in 1-phenyloctane (*C*_0_ = 0.9 mM, dashed black curve and solid red curve, respectively), immediately after 60 s of 367 nm UV irradiation (solid blue curve), and after another 15 min in the dark (dashed red curve). **c** Time evolution of *I*_*DS*_ (at *V*_GS_ = 0 V) before, during and after UV irradiation. The purple area in the graph corresponds to the duration of UV light irradiation. The inset shows the evolution of the MC-induced extra current Δ*I* during UV light irradiation (blue squares) and the applied monoexponential fit (red line) to deduce the corresponding time constant *τ*_hν,E_. We highlight that the time constants *τ*_hν,O_ and *τ*_hν,E_ derived by the optical and electrical experiments are characteristic of different physical phenomena, being *τ*_hν,O_ associated with the isomerization in solution, and *τ*_hν, E_ with the formation of the self-assembly. **d** Time evolution of *I*_DS_ during consecutive irradiation cycles with the same irradiation time; **c**–**d** Measurements performed on commercial CVD graphene devices and concentration *C*_0_ = 4 mM. **e** Thermal relaxation of MC monolayer: comparison between the time evolution of the current *I*_DS_ and the absorbance Abs_590nm_ (*C*_0_ = 2 mM) as well as the substrate coverage, as extracted from STM images (*C*_0_ = 1.8 mM).

Our approach to employ graphene devices as highly sensitive detector to obtain greater insight into the self-assembly dynamics is illustrated in Fig. 3a. We covered a graphene field-effect transistor with a small drop of SP solution, and triggered the formation/desorption of the metastable MC assembly at the graphene/solution interface by switching UV light on and off. Due to the ultrahigh surface sensitivity of graphene, the MC adlayer induces a change in its electrical conductance, allowing us to monitor the dynamics of formation (desorption) of the self-assembled adlayer by recording the electrical current flowing through graphene during (after) UV light irradiation.

To investigate how the transfer curve of graphene is modified by the formation of the MC assembly, we employed a field-effect transistor based on mechanically exfoliated single layer graphene. The pristine device displayed an almost ideal transfer curve with a charge neutrality point at *V*_G_ = 2 V (Fig. 3b, black dashed trace). Covering its surface with a 6 μL drop of SP solution resulted in a relatively small shift of the charge neutrality point towards a more negative value, *V*_G_ = −2 V (Fig. 3b, red trace). Irradiation with UV light, triggering formation of the MC assembly, largely modified the transfer characteristics, inducing a significant additional shift of the charge neutrality point to *V*_G_ = −11 V (Fig. 3b, blue trace), indicating n-type doping. As a control experiment, irradiation of a device covered by 1-phenyloctane without SP molecules introduces only a minor change in its electrical characteristics (Supplementary Fig. 3).

The origin of the shift in the charge neutrality point induced by the formation of the MC adlayer can be understood on the basis of the nanoscale molecular arrangement^26^. In particular, the graphene area covered by the MC adlayer experiences a field effect similar to that introduced by a gate terminal, generated by orderly aligned molecular dipoles with an out-of-plane component. In this scenario, the electrical current changes linearly with the surface coverage, and the maximum shift in the charge neutrality point is reached when the entire graphene surface is covered by the self-assembled MC adlayer (see Supplementary discussion 1). After 15 min, the *I*_DS_–*V*_G_ characteristics recovered and almost completely resembled those of the initial SP solution on graphene, indicating dissolution of the MC assembly (Fig. 3b, red dashed trace). The fact that the effect on the electrical characteristics can be rationalized on the basis of the evolution of the nanoscale arrangement further confirms the formation and dissolution of the ordered MC assembly at the graphene/solution interface.

To track in real time the dynamics of formation and desorption of the self-assembled adlayer, we monitored the drain source current *I*_DS_ while UV light was switched on and off (Fig. 3c). In order to quantitatively compare the results obtained with different devices, we employed commercial devices based on CVD graphene with the same width/length (*W*/*L*) ratio. Representative measurements performed in a device with *W* = *L* = 50 µm are shown in Fig. 3c. We interpreted the *I*_DS_ change as the result of the adsorption and desorption of the MC adlayer, which acted as a light-induced gate terminal, with an amplitude proportional to the number of MC molecules on the graphene surface (see Supplementary discussion 2). In good agreement with the measurement in Fig. 3b, we found that *I*_DS_ increased steeply during the initial stage of UV irradiation due to the formation of the MC adlayer, which progressively covers the graphene surface. After ~30 s of irradiation, *I*_DS_ reached a plateau, which corresponds to a full coverage of the graphene surface by the MC monolayer. When the UV light was turned off, the current decreased and eventually reached the preirradiation value, following the desorption of the MC adlayer.

We found the variation Δ*I* = *I*_DS_ (*t*) − *I*_0_, where *I*_0_ is the current measured before UV irradiation (inset in Fig. 3c), to be quantitatively consistent in three devices characterized by a different area of the graphene active channel, with the same *W*/*L* ratio, but different *W* and *L* (*W* = *L* = 50 µm and *W* = *L* = 100 µm, see Supplementary Figs. 4–6 and Supplementary Table 2). Indeed, the intrinsic parameter which is modified by the formation of the MC assembly is the resistance of graphene, which depends on the *W*/*L* ratio, but not on the absolute value of *W* and *L*. In principle, by scaling down the area of the graphene active channel to *W* = *L* = 100 nm, which is within the reach of standard e-beam lithography, one could detect the dynamics of self-assembly in a region as small as that investigated by STM (Fig. 2d), while maintaining the same Δ*I* recorded for a device characterized by a six-orders-of-magnitude-larger active area (*W* = *L* = 100 µm).

Detailed analysis reveals that the increase in Δ*I* during UV light irradiation can be fitted by the monoexponential equation Δ*I* (*t*) = Δ*I*_sat_ − *B* × exp (−*t*/*τ*_*hν*,E_), as can be understood using a simple model (see Supplementary discussion 2). In particular, in the case shown in Fig. 3c, we determined an electrical time constant *τ*_*hν*,E_ = 6 s, being markedly different from the optical time constant extracted for the photoisomerization in solution. Indeed, both time constants are associated to very different processes, one reflecting the photoisomerization in the bulk solution and the other one the formation of the MC self-assembly at the solid/liquid interface. We highlight that the same power density and the same UV light source were used for all experiments carried out in this study (see “Methods”).

Monitoring the change in current during subsequent cycles in which UV irradiation was repeatedly switched on and off at regular time intervals, while keeping *V*_G_ = 0 V, reveals that evolution of the current was not only qualitatively analogous, but also quantitatively. In the four consecutive cycles shown in Fig. 3d, the average saturation value reached by the current is *I*_DS_ = 6.05 µA, with a deviation of only 15 nA. Moreover, in each cycle the current value reached after thermal relaxation is very similar to the preirradiation value, resulting *I*_DS_ = 3.05 ± 0.02 µA. In this way, the current increase Δ*I* upon successive cycles of UV irradiation was within 1% (Δ*I* = 3.00 ± 0.02 µA). Even the time evolution of *I*_DS_ recorded in three subsequent irradiation cycles with different irradiation time was found to overlap closely (Supplementary Fig. 7).

A different situation is encountered for the dynamics of desorption of self-assembly. Figure 3e shows the evolution of Abs_590nm_ and *I*_*DS*_ after 60 s the UV irradiation, and compares them with the areal coverage extracted from the STM movie in Supplementary Fig. 2. We found that Abs_590nm_ could be fitted by a simple exponential decay in the from Abs_590nm_ (*t*) = Abs_sat_ × exp (−*t*/*τ*_Δ,O_), where *τ*_Δ,O_ = 87 s denotes the time constant for the thermal decay in solution. Instead, the desorption of the self-assembly as revealed by *I*_DS_ follows a more complex dynamic, which can be fitted by the sum of two exponential decays characterized by different time constants (see Supplementary Fig. 8). Through STM imaging, it is possible to follow how the self-assembled adlayer evolves during successive scans in the same area, initially covering the whole substrate surface and desorbing rather abruptly after ~6 min.

While Abs_590nm_ is related to the thermal relaxation of MC in solution, the decay of *I*_DS_ and the variation in surface coverage measured by the STM after UV irradiation describe the same physical phenomenon, which is the desorption of the MC self-assembly. However, the two techniques probe a different number of molecules and possess a different time resolution. In particular, the STM images shown in Fig. 2 monitor the dynamics of ~3800 molecules, as determined by the nominal size of the image (90 × 90 nm^2^) and considering the size of the unit cell. Therefore, the spatially confined nature of STM impedes its use for an accurate description of the dynamics of a large population of molecules. For instance, the first STM image acquired after UV irradiation in another area of the sample displayed a self-assembled adlayer, which did not cover completely the scanned area (see Supplementary Fig. 2). Instead, the time resolution is limited by the duration of image acquisition, which is *t* = 34 s for the measurements in Fig. 2. Importantly, it remains unclear whether and to which extent the presence of the STM tip perturbs the dynamics of desorption. In contrast, our electrical detection of the self-assembly is noninvasive, and monitors the dynamics of a molecular ensemble of more than 4 × 10^9^ molecules, covering an area as large as 100 × 100 µm^2^. Self-assembly, which is an ensemble process characterized by relatively slow kinetics evolving on the time scale of seconds, is perfectly suited to be monitored with conventional electronics, which grants a typical sampling rate of 10–100 Hz. In our experiment, the time resolution was set to 100 ms, which was ideal to capture the few-seconds dynamics of our system (see Supplementary discussion 3).

### Concentration dependence of the self-assembly dynamics

**Fig. 4:**
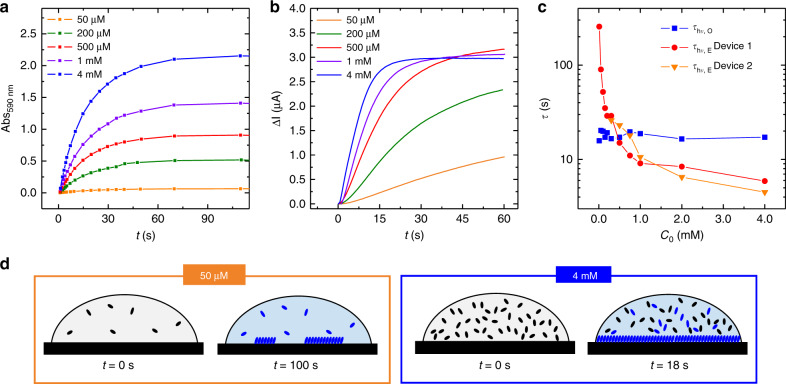
Concentration dependence of photoisomerization and self-assembly dynamics. **a**, **b** Time evolution of absorbance (measured at *λ* = 590 nm) and electrical current at different initial SP concentrations *C*_0_. **c** time constants *τ*_hν,O_ and *τ*_hν,E_ extracted from the absorbance variation of a solution (photoisomerization) and from the variation of the electrical current (formation of the self-assembled adlayer). The measurements performed in two devices are shown, one with *L* = *W* = 50 µm and another one with *L* = *W* = 100 µm. **d** Cartoons depicting our interpretation of the measured data. At low initial concentration, the graphene surface is not covered by a continuous adlayer even after a relatively long irradiation time, after the stationary state is reached (*t* = 100 s); at high initial concentration, the graphene surface is fully covered before the stationary state is reached (*t* = 18 s).

Finally, the potential of graphene devices to monitor in real-time molecular processes can be fully appreciated by comparing the dynamics extracted for the formation of the self-assembly and the photoisomerization at different concentrations *C*_0_. Figure 4a, b display the time evolution of Abs_590nm_ and of Δ*I*, which reflect the kinetics of SP photoisomerization in the bulk solution and of formation of the MC self-assembled adlayer, respectively. Switching on the UV light (at *t* = 0 s) results in an abrupt increase in both Abs_590nm_ and Δ*I*, which results from the photogeneration of MC molecules and by the gating effect of the MC adlayer at the graphene surface. While Abs_590nm_ reaches a different saturation value in all cases, Δ*I* does not reach a saturated value at low concentration (*C*_0_ < 0.75 mM), while it does at high concentration (*C*_0_ > 0.75 mM). In addition, while the saturated value reached by Abs_590nm_ increases with *C*_0_, the saturated value reached by Δ*I* is similar (for *C*_0_ > 0.75 mM), indicating that the maximum gating effect is the same at the different concentration. Based on our data we conclude that after 1 min of UV irradiation a complete self-assembled adlayer is formed whenever Δ*I* reaches saturation, i.e., for *C*_0_ > 0.75 mM. We stress that Δ*I* was quantitatively similar in the three devices, which were tested with multiple *C*_0_ (Supplementary Figs. 4–6).

Another important difference emerges by comparing the time constants *τ*_hν,O_ and *τ*_hν,E_, extracted by fitting Abs_590nm_ and Δ*I* with a monoexponential equation, as described above (Fig. 4c). *τ*_hν,O_, associated to the photoisomerization in the bulk solution, does not vary significantly in the studied range, remaining in the range *τ*_hν,O_ = 18 ± 2 s. On the contrary, *τ*_hν,E_, associated to the formation of the MC assembly, depends dramatically on the initial SP concentration, decreasing steadily in the whole concentration range. The same trend was extracted in three different devices (for clarity, only two are shown in Fig. 4c). We estimate that the dynamics of formation of the monolayer cover a range from above 200 s at 10 µM to 6 s at 4 mM (see Supplementary information). This variation suggests that the formation of the self-assembly requires a critical concentration of MC molecules in the vicinity of the surface. At low SP concentration, such concentration is not achieved even when the stationary state is reached upon irradiation of the solution (Fig. 4d). Instead, above 0.75 mM the saturation in the current is reached before the stationary state in solution, indicating that the concentration of MC is above the critical value needed to generate a monolayer (Fig. 4d). For example, at 4 mM the formation of self-assembly is characterized by a time constant *τ*_hν,E_ = 6 s. Assuming that the self-assembly covers completely the surface after a time 3*τ*_hν,E_ = 18 s, from the spectroscopic data shown in Fig. 4a, it is possible to see that the MC concentration is ~60% obtained at the stationary state. In this way, we demonstrate the ability to precisely disentangle the dynamics of very different molecular processes occurring simultaneously thanks to the ultrahigh surface sensitivity of graphene.

In conclusion, we have shown that graphene devices constitute a robust experimental platform to monitor in real time the complex dynamic process of molecular self-assembly at the solid/liquid interface. As a proof of concept, we demonstrated the characterization of the dynamics of formation and desorption of a metastable self-assembled adlayer formed by an SP derivative at the graphene/solution interface. Such a precise monitoring of an interfacial phenomena is not accessible via bulk characterization techniques. The electrical read out is highly sensitive, practical, reliable, and in principle offers an ultrafast time response, making it suitable to follow rapidly evolving processes. While in this work the temporal resolution is limited by our experimental setup, ultrafast graphene devices operating above 400 GHz range have been demonstrated^32^ and offer the opportunity to follow molecular dynamics in the few-nanoseconds range. We anticipate that our strategy will be exploited to resolve the dynamics of more complex molecular ensemble processes taking place on solid surfaces, such as the formation of 2D polymers^33^. It also holds great potential for applications in (bio)diagnostic and (bio)chemical sensing.

## Methods

### Synthesis

The synthesis of the SP derivative^26^ is detailed in the Supplementary note 1.

### Optical characterization

All the experiments reported here were performed using 1-phenyloctane as solvent, purchased from TCI Chemicals and used without further purification. Importantly for our experiments, the absorbance of 1-phenyloctane is negligible at 365 nm. UV light irradiation was performed with optical fiber-coupled LEDs (ThorLabs), *λ*_max_ = 367 nm, FWHM = 7 nm. For optical and electrical experiments, collimated UV irradiation light intensity was carefully controlled (*P*_d_ = 0.5 mW cm^−2^, measured with photodiode power meter, ThorLabs), in order to obtain reproducible conditions for the kinetic measurements. Such relatively low power density was employed to avoid an unintentional increase in the droplet temperature during the few-minutes irradiation. Indeed, the realization of a stable stationary state in the optical characterization rules out a significant temperature increase, as discussed in Fig. S1.

Optical micrographs highlighting the color change of SP solution were recorded using a stereomicroscope equipped with a digital camera (Olympus SZ61TR). UV–Vis absorption spectra and isomerization kinetics were recorded at room temperature (26 °C) with a Jasco V650 spectrophotometer, in matched quartz Suprasil cuvettes (Helma). Absorption spectra were recorded in 10 mm optical path cuvettes. In order to reproduce the droplet-on-device conditions, isomerization kinetics were recorded by monitoring the absorbance at 590 nm (A_590nm_) of unstirred solutions in 1 mm optical path cuvettes. We chose 590 nm and not the max (ca. 605 nm) in order to avoid reaching too high absorbance values in the experiments at the highest concentrations. For consistency we performed all measurements at the same wavelength.

For photoisomerization kinetics, irradiation of the solution was performed in situ within the spectrophotometer chamber, by shining a collimated beam of UV light on the front face of the cuvette, tilted of ca. 30° with respect to the spectrophotometer’s analysis beam. Starting from thermal equilibrium conditions (A_59nm_~0—negligible amount of MC in solution) UV irradiation was performed for fixed amounts of time (*t* = 1, 2, 3, 4, 5, 7, 10, 15, 20, 25, 30, 35, 40, 50, 70, 110, 150 s), and A_590nm_ was recorded immediately after switching off UV light.

### Scanning tunneling microscopy

Investigation on the self-assembly of SP and MC was performed by STM at ambient pressure and room temperature, using freshly cleaved highly oriented pyrolytic graphite (HOPG) as substrate. The experiments were performed using a Veeco Multimode III (Bruker) equipped with an STM head and a 1 µm-range piezoelectric scanner (A-Piezo, Bruker), working in constant current mode. STM tips were mechanically cut from a Pt/Ir (80:20) wire (0.25 mm diameter, Goodfellow). Self-assembly was studied at the solid–liquid interface between HOPG and a supernatant solution, by applying 6 µL of the latter on the substrate, after having checked the integrity of substrate and tip by visualizing the graphite lattice. The raw STM data were processed using a dedicated image processing software (Gwyddion), by means of flattening and subtraction of a 2° polynomial background. In Fig. 2b, the drift of the piezo was corrected using the underlying graphite lattice as a reference. The lattice of the underlying substrate was visualized by lowering the bias voltage *V*_t_ to 10 mV after imaging the MC assembly and increasing the tunneling current to *I*_t_ = 60 pA. Tip height and current were measured for all STM images. Unit cell parameters were obtained by Fourier analysis.

STM experiments were performed at an initial *C*_0_ = 1.8 mM concentration of SP. UV light irradiation experiments were performed in situ, by irradiating the solution on the substrate after having verified the absence of any supramolecular packing of SP prior to light irradiation. The experimental setup to perform UV (365 nm) irradiation involved a UV lamp (*P*_d_~2.0 mW cm^−2^), and was performed by directly placing the lamp close (*d* < 5 cm) to the sample, after stopping the STM scanning, but keeping the tip in tunneling contact.

STM imaging was subsequently performed in the same sample area, permitting to visualize the evolution of the MC adlayer. For this experiment, the time resolution is limited by the time necessary to acquire successive frames by STM ~30 s. Also, while we could use STM to monitor how the self-assembly is desorbed, we were not able to acquire consecutive high-resolution images for the formation of the self-assembly. Indeed, the UV irradiation necessary to trigger the formation of the MC assembly introduces a high level of noise in the STM measurements, which prevented the acquisition of images.

### Device fabrication and characterization

Back-gated devices based on scotch-tape exfoliated or commercial CVD graphene were employed in this work. The scotch-tape devices were fabricated on SiO_2_ (90 nm)/Si substrates with a Microtech laser writer, equipped with a 405 nm laser standard photoresist (AZ1505, Microchemicals). A 35-nm-thick Au film (without adhesion layer) was thermally evaporated onto the patterned photoresist and lift-off was carried out in warm acetone (40 °C). Commercial chips containing 36 field-effect transistors based on CVD graphene were employed (GFET S20 from Graphenea, SiO_2_(90 nm)/Si n++ dielectric_,_ Ni/Al electrodes). Before performing the measurements, the devices were cleaned by immersion in acetone and N-methyl-2-pyrrolidone, and then rinsed with isopropanol. With this cleaning procedures, the devices with *L* = *W* = 50 µm and *L* = *W* = 100 µm showed almost ideal graphene characteristics, with a charge neutrality point close to *V*_G_ = 0 V. Upon casting a droplet of SP solution in 1-phenyloctane, the charge neutrality point shifted to *V*_G_ < 0 V, so that the irradiation experiments carried out at *V*_G_ = 0 V probed a linear part of the transfer characteristics, in which current increase corresponded to a shift of the charge neutrality point to more negative values (n-type doping).

Irradiation experiments on the graphene field-effect transistors were performed at room temperature (26 °C), by shining a beam of collimated UV light perpendicularly to the surface of the device covered with a droplet of 1-phenyloctane solution of SP and recording the current while applying a drain–source voltage *V*_DS_ = 10 mV. Up to three devices on the same chip and covered by the same solution droplet could be measured simultaneously during the same UV irradiation. The conditions for UV irradiation were the same employed for the optical characterization (fiber-coupled LEDs ThorLabs, *λ*_max_ = 367 nm, FWHM = 7 nm, *P*_d_ = 0.5 mW cm^−2^). Experiments were performed by progressively increasing the initial SP concentration (*C*_0_), starting from pure solvent, upon adding small amounts (~few microlitre) of concentrated solutions adjusted to obtain the desired concentration. For the highest concentrations employed in this study (2 and 4 mM), we casted the lowest volume of solution capable of covering all the devices under test (8 µl). In this way, we minimized the thickness of the droplet, and therefore the optical path and the internal filter effect (see also Supplementary Fig. 1). Intervals of 20 min between the additions and the measurements were allowed, in order to obtain homogeneous concentration of SP by simple diffusion.

## Supplementary information

Supplementary Information

## Data Availability

The data that support the findings of this study are available from the corresponding authors upon reasonable request.

## References

[1] Barth JV, Costantini G, Kern K (2005). Engineering atomic and molecular nanostructures at surfaces. Nature.

[2] De Feyter S, De Schryver FC (2005). Self-assembly at the liquid/solid interface: STM reveals. J. Phys. Chem. B.

[3] Mali KS, Pearce N, De Feyter S, Champness NR (2017). Frontiers of supramolecular chemistry at solid surfaces. Chem. Soc. Rev..

[4] Love JC, Estroff LA, Kriebel JK, Nuzzo RG, Whitesides GM (2005). Self-assembled monolayers of thiolates on metals as a form of nanotechnology. Chem. Rev..

[5] Gobbi M, Orgiu E, Samorì P (2018). When 2D materials meet molecules: opportunities and challenges of hybrid organic/inorganic van der waals heterostructures. Adv. Mater..

[6] Rabe JP, Buchholz S (1991). Commensurability and mobility in two-dimensional molecular patterns on graphite. Science.

[7] Fang Y (2016). Dynamic control over supramolecular handedness by selecting chiral induction pathways at the solution–solid interface. Nat. Chem..

[8] Wasio NA (2014). Self-assembly of hydrogen-bonded two-dimensional quasicrystals. Nature.

[9] Urgel JI (2016). Quasicrystallinity expressed in two-dimensional coordination networks. Nat. Chem..

[10] De Feyter S, Gesquiere A, Klapper M, Müllen K, De Schryver FC (2003). Toward two-dimensional supramolecular control of hydrogen-bonded arrays: the case of isophthalic acids. Nano Lett..

[11] Ciesielski A (2014). Dynamic covalent chemistry of bisimines at the solid/liquid interface monitored by scanning tunnelling microscopy. Nat. Chem..

[12] Xue Y, Zimmt MB (2012). Patterned monolayer self-assembly programmed by side chain shape: four-component gratings. J. Am. Chem. Soc..

[13] Song W, Martsinovich N, Heckl WM, Lackinger M (2013). Born–Haber cycle for monolayer self-assembly at the liquid–solid interface: assessing the enthalpic driving force. J. Am. Chem. Soc..

[14] Gutzler R, Cardenas L, Rosei F (2011). Kinetics and thermodynamics in surface-confined molecular self-assembly. Chem. Sci..

[15] Palma C-A, Cecchini M, Samorì P (2012). Predicting self-assembly: from empirism to determinism. Chem. Soc. Rev..

[16] Chen J (2018). Building two-dimensional materials one row at a time: avoiding the nucleation barrier. Science.

[17] Li H (2020). Nucleation–elongation dynamics of two-dimensional covalent organic frameworks. J. Am. Chem. Soc..

[18] Stabel A, Heinz R, De Schryver FC, Rabe JP (1995). Ostwald ripening of two-dimensional crystals at the solid–liquid interface. J. Phys. Chem..

[19] Samorí P, Müllen K, Rabe JP (2004). Molecular-scale tracking of the self-healing of polycrystalline monolayers at the solid–liquid interface. Adv. Mater..

[20] Eder G (2011). Incorporation dynamics of molecular guests into two-dimensional supramolecular host networks at the liquid–solid interface. Langmuir.

[21] Bhattarai A, Mazur U, Hipps KW (2014). A single molecule level study of the temperature-dependent kinetics for the formation of metal porphyrin monolayers on Au(111) from solution. J. Am. Chem. Soc..

[22] Yokoyama S, Hirose T, Matsuda K (2014). Phototriggered formation and disappearance of surface-confined self-assembly composed of photochromic 2-thienyl-type diarylethene: a cooperative model at the liquid/solid interface. Chem. Commun..

[23] Yokoyama S, Hirose T, Matsuda K (2015). Effects of alkyl chain length and hydrogen bonds on the cooperative self-assembly of 2-thienyl-type diarylethenes at a liquid/highly oriented pyrolytic graphite (HOPG) interface. Chem.—Eur. J..

[24] Maeda N, Hirose T, Yokoyama S, Matsuda K (2016). Rational design of highly photoresponsive surface-confined self-assembly of diarylethenes: reversible three-state photoswitching at the liquid/solid interface. J. Phys. Chem. C.

[25] Bonacchi S (2015). Surface-induced selection during in situ photoswitching at the solid/liquid interface. Angew. Chem.—Int. Ed..

[26] Gobbi M (2018). Collective molecular switching in hybrid superlattices for light-modulated two-dimensional electronics. Nat. Commun..

[27] Henß A-K (2019). Density fluctuations as door-opener for diffusion on crowded surfaces. Science.

[28] Guan J (2018). Direct single-molecule dynamic detection of chemical reactions. Sci. Adv.

[29] Xu S (2017). Real-time reliable determination of binding kinetics of DNA hybridization using a multi-channel graphene biosensor. Nat. Commun..

[30] Klajn R (2014). Spiropyran-based dynamic materials. Chem. Soc. Rev..

[31] Flannery JB (1968). Photo- and thermochromic transients from substituted 1’,3’,3’-trimethylindolinobenzospiropyrans. J. Am. Chem. Soc..

[32] Cheng R (2012). High-frequency self-aligned graphene transistors with transferred gate stacks. Proc. Natl. Acad. Sci. USA.

[33] Grill L, Hecht S (2020). Covalent on-surface polymerization. Nat. Chem..

