# Supplementary material for: Graphene transistors for real-time monitoring molecular self-assembly dynamics
Source: Nat Commun. 2020 Sep 18;11:4731. doi: 10.1038/s41467-020-18604-4 (PMC7501237; doi:10.1038/s41467-020-18604-4)
Supplement: Supplementary file 1 — Supplementary Information [file 41467_2020_18604_MOESM1_ESM.pdf]

Supplementary information

Graphene transistors for real-time monitoring molecular self-assembly dynamics

Gobbi et al.

Table of contents

Supplementary Figure 1. .... S2
Supplementary Figure 2. .... S4
Supplementary Figure 3. .... S5
Supplementary Figure 4. .... S6
Supplementary Figure 5. .... S8
Supplementary Figure 6. .... S10
Supplementary Figure 7. .... S11
Supplementary Figure 8. .... S12
Supplementary Table 1.. S13
Supplementary Table 2.. S14
Supplementary Note 1.. S15
Supplementary Discussion 1 .... S17
Supplementary Discussion 2 .... S19
Supplementary Discussion 3 .... S22
Supplementary References.. S23

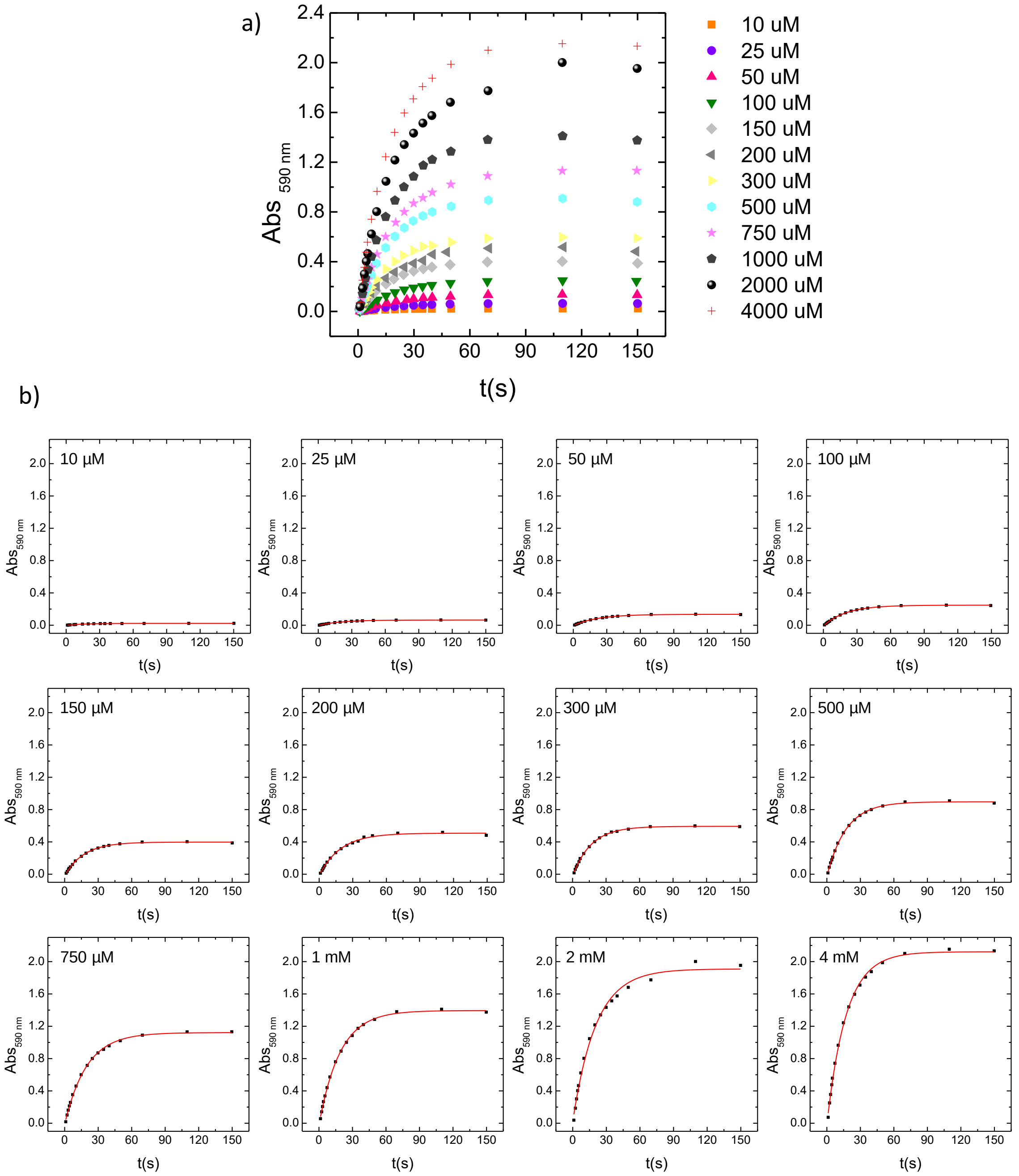

Supplementary Figure 1: Time evolution of the absorbance of SP/MC solutions at different concentrations upon UV irradiation. (a) Absorbance measured at 590 nm for different initial spiropyran concentrations C_0 upon 365 nm UV irradiation. Each data point is collected immediately after irradiating the solution for a fixed amount of time. (b) Fit of the time evolution of absorbance with the equation $Abs_{590\ nm}(t) = Abs_{sat} - B \times \exp(-t / \tau_{h\nu,O})$. Black dots are experimental data, red curves the fitting curves. The values extracted from the fit for Abs_{sat}, B and $\tau_{h\nu,O}$ are listed in Supplementary Table 1.

In all the curves, a stationary state is reached in which the photo-induced SP → MC isomerization is balanced by the thermal MC → SP isomerization. We note that at high concentration (C_0 > 0.75 mM), the increase in the absorbance at the stationary state does not scale linearly with the concentration. This is due to due to the inner filter effect related to the high absorption of UV light by SP at high concentration, when most UV photons are absorbed by the SP molecules in the region facing the UV light, preventing the UV irradiation to penetrate further in the bulk solution. We highlight that this effect is less significant for the irradiation of the droplet on the devices. Indeed, for the highest concentrations we employed a 8 µL droplet, which covered a roughly circular area with a diameter of approximately 5 mm. Therefore, we estimate the thickness of the droplet in the highest concentration case was approximately 0.4 mm, significantly shorter than the 1-mm optical path of the cuvette.

We also highlight that the presence of such stationary state indicates that the UV light irradiation does not introduce a significant increase in the solution temperature. In particular, if the solution were heating up significantly during irradiation, one should expect a progressively more important contribution of the thermally activated MC → SP recovery with respect to the constant photo-isomerization, effectively increasing the SP fraction over time. Instead, our data do not show any increase in the SP content after the stationary state is reached, as the absorption at 560 nm does not decrease after 3 minutes of irradiation, demonstrating a minor effect of temperature at least for the duration of our irradiation.

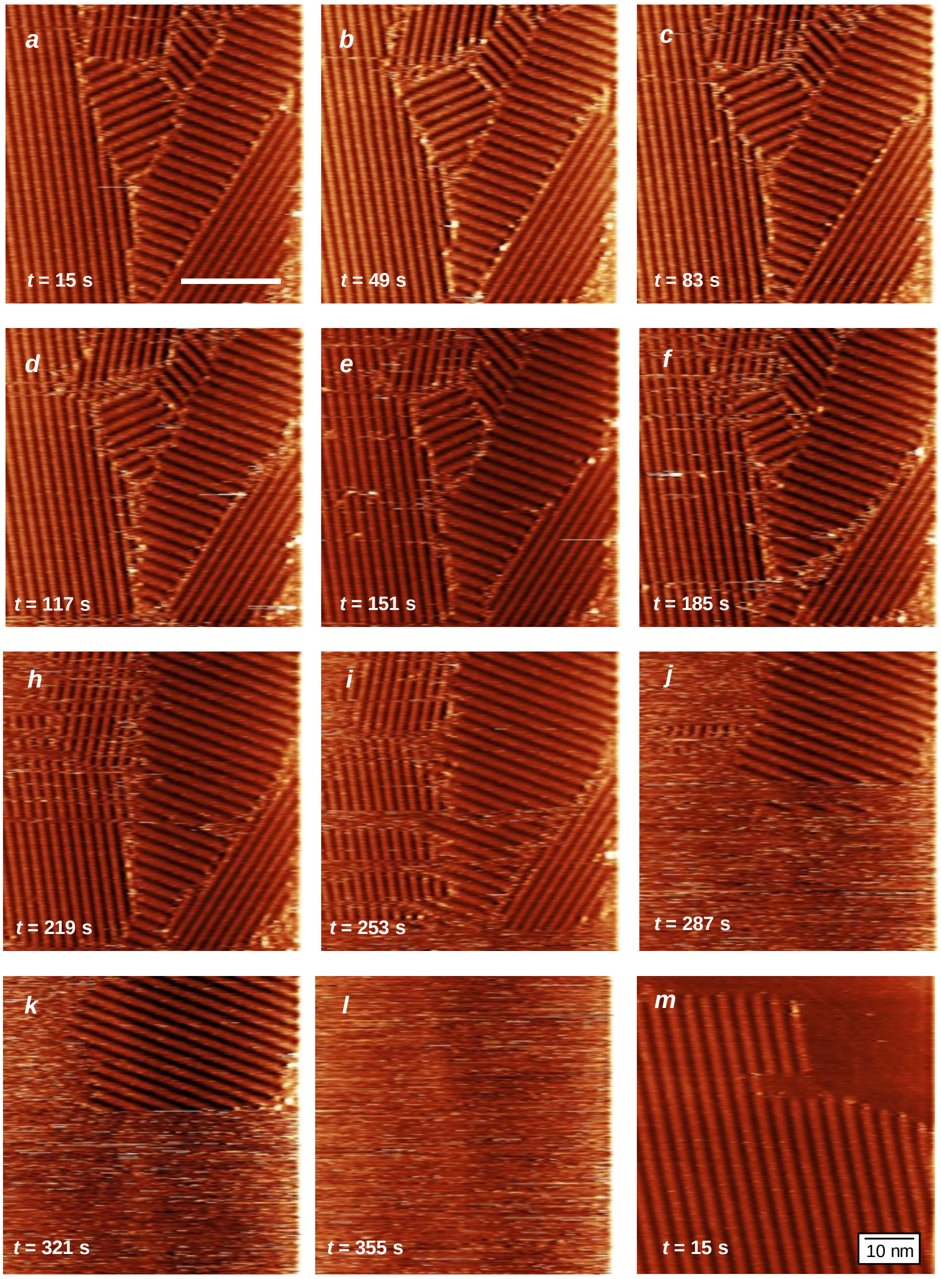

Supplementary Figure 2: Evolution of the merocyanine assembly imaged by Scanning Tunneling Microscopy. (a-l) The images were acquired subsequently in the same location. Before acquisition, a 6 µL drop of spiropyran solution in 1-phenyloctane (C_0 = 1.8 mM) was casted onto the highly oriented pyrolytic graphite substrate and irradiated with UV light during 6 s. The first image was started 15 s after switching off the UV light, each image was acquired in 34 s. (m) In another experiment, the first image did not show a complete self-assembled adlayer, highlighting how the images in (a-l) cannot be used to describe the evolution of the assembly over the whole substrate. Tunneling Current I_T = 60 pA, Tunneling Voltage V_T = 600 mV; (d) I_T = 20 pA, V_T = 600 mV.

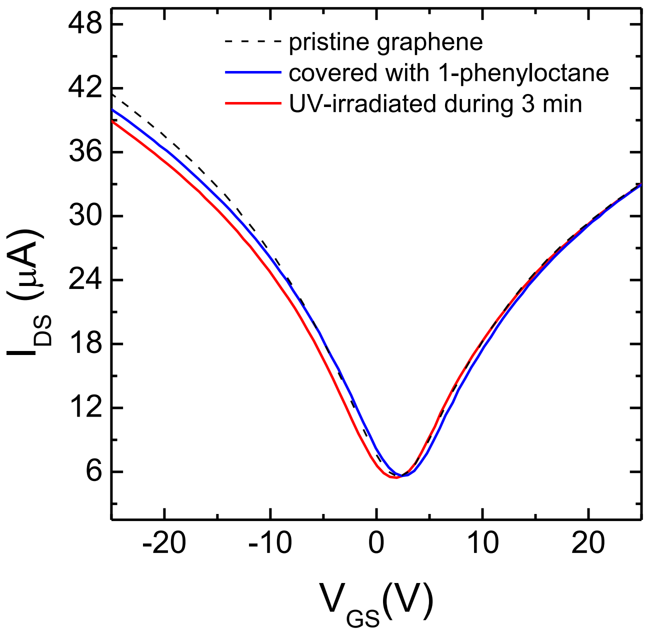

Supplementary Figure 3: Effect of the UV irradiation on a device covered by 1-phenyloctane. Drain current I_{DS} versus gate voltage V_{GS} measured for a clean graphene device (black dashed trace), after casting a 6 µl droplet of 1-phenyloctane without SP/MC molecules (blue trace), and after three minutes of UV irradiation (red trace). While the presence of 1-phenyloctane does not change significantly the graphene characteristics, UV irradiation introduces a minor shift ($\Delta V_{CNP} < 1$V) in the charge neutrality point. For this measurement, the sample was irradiated during three minutes, using the same power employed for triggering the SP→ MC isomerization ($_{Pd}$ = 0.5 mW cm^{-2}). We ascribe the origin of the recorded minor shift to a UV-induced dissolution of molecular adsorbates that could be present as a contamination on the graphene surface. A similar explanation was put forward to explain the doping observed in bare graphene irradiated by UV light[1]. We highlight that the shift recorded in this case is approximately one tenth of that recorded for the same device when covered by the SP solution (shown as Fig. 3a), and it was neglected for the analysis of the dynamics.

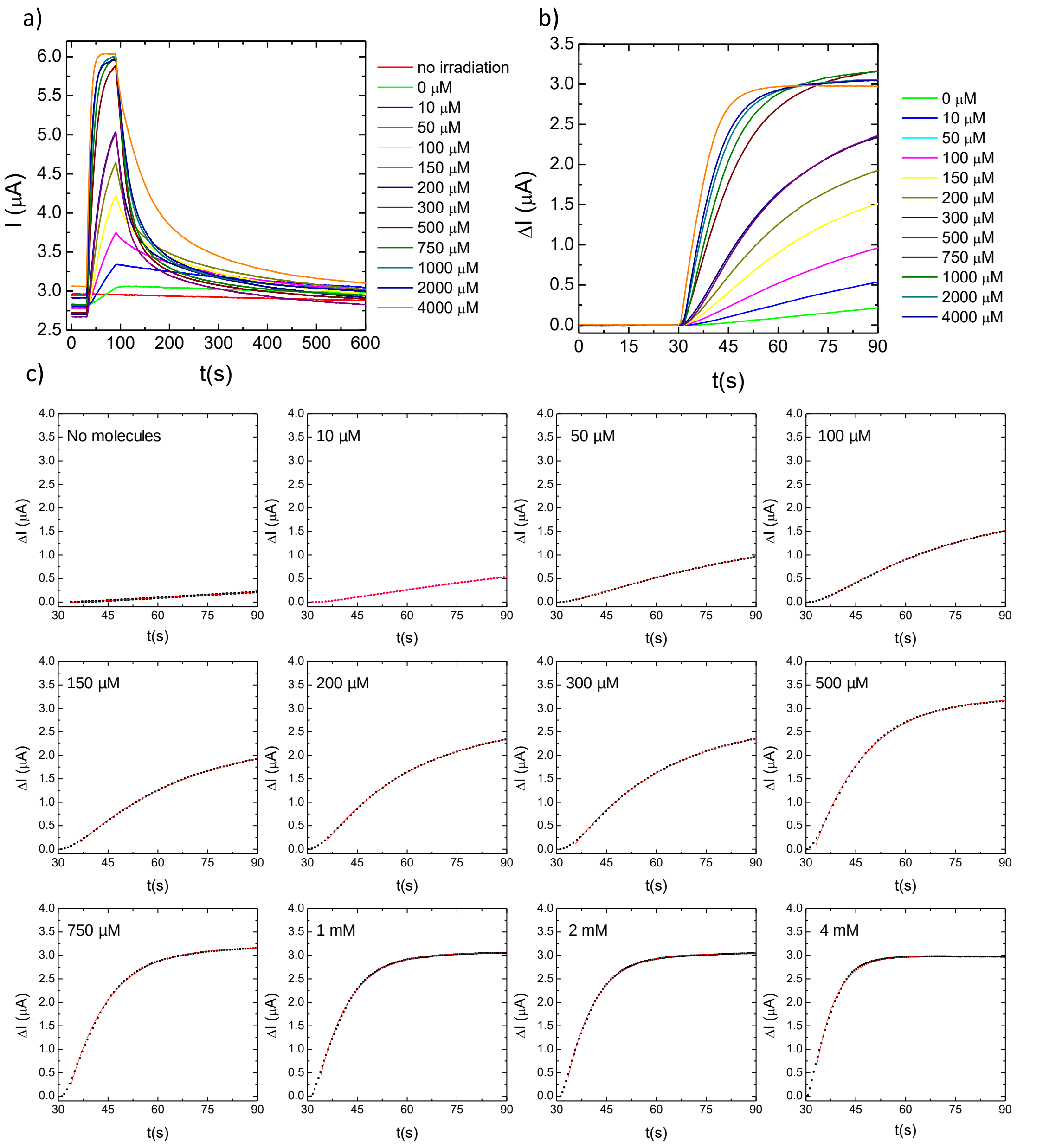

Supplementary Figure 4: Time evolution of the electrical current in a graphene device at different C_0. (a) Time evolution of current $I_{DS}(t)$ measured during subsequent irradiation/relaxation cycles measured in a device (hereafter D1) covered by solutions with different SP concentration. For all measurements, UV light was switched on at t = 30 s and off at t = 90 s. (b) $\Delta I\ (t) = I_{DS}\ (t) - I_0$ measured during irradiation at different C_0, where $I_{DS}(t)$ is shown in (a) and I_0 is the current before irradiation. This set of measurement is presented in the main text. (c) Fit of $\Delta I\ (t)$ to the equation $\Delta I\ (t) = \Delta I_{sat} - B \times \exp(-t / \tau_{h\nu,E})$. Black dots are experimental data, red lines are the fitting curves. The curves were fitted starting approximately 7 s after switching on the UV irradiation. During such initial time, a finite MC concentration

builds up, triggering the subsequent self-assembly process (see also Supplementary Discussion 2). The values extracted from the fit for ΔI_{sat}, B and $\tau_{h\nu,E}$ are listed in Supplementary Table 2. We highlight that the minor ΔI recorded for the top left panel in (c) corresponds to a situation in which graphene is covered only by the solvent (1-phenyloctane), see also Fig. S3. Channel size: $L = W = 50$ µm. Bias voltage $V_{DS} = 10$ mV; $V_{GS} = 0$ V.

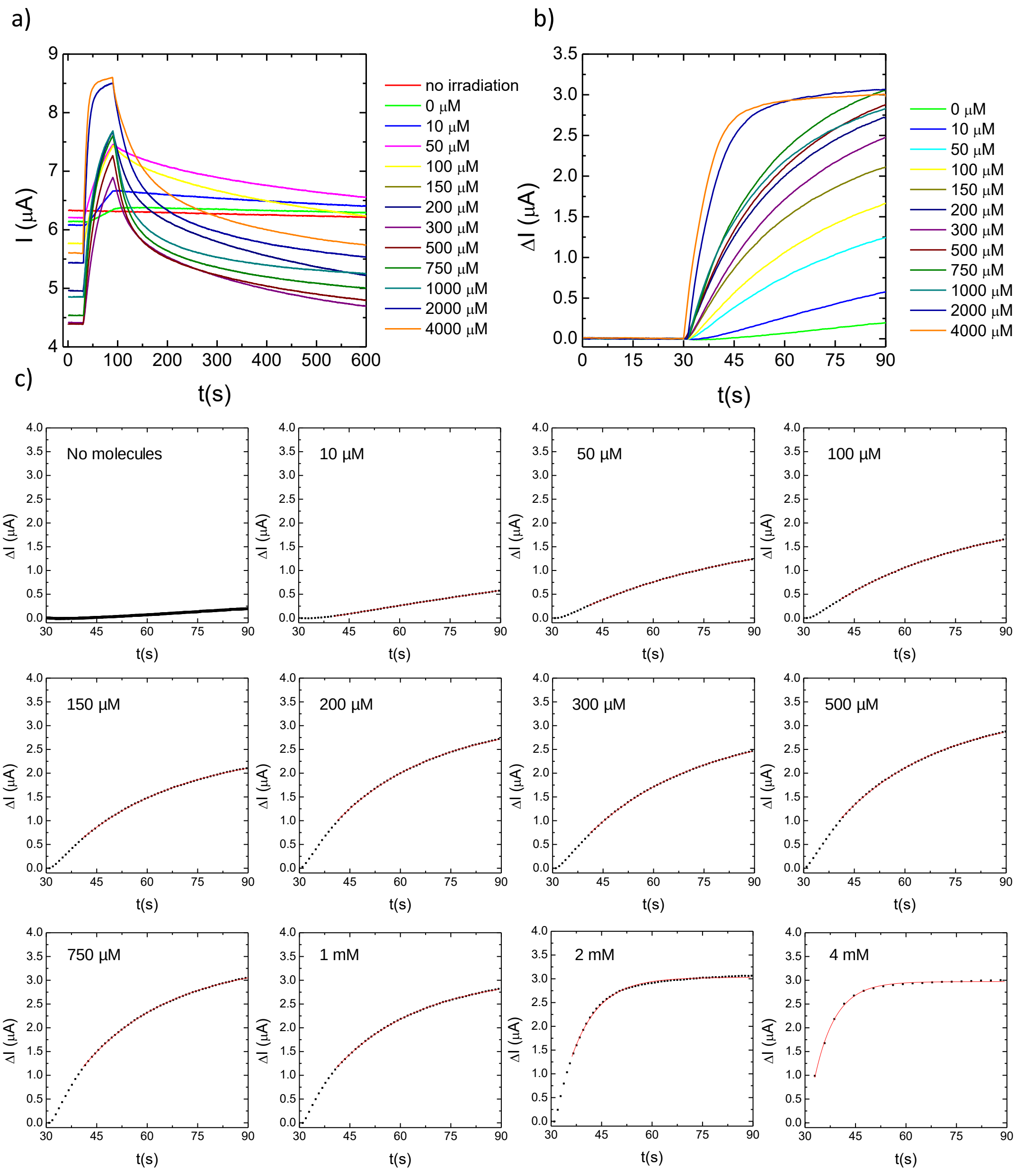

Supplementary Figure 5: Time evolution of the electrical current for a second device D2 at different C_0. (a) Time evolution of current I_{DS} (t) measured during subsequent irradiation/relaxation cycles for another device (hereafter D2) covered by solutions with different SP concentration. For all measurements, UV light was switched on at t = 30 s and off at t = 90 s. (b) $\Delta I\ (t) = I_{DS}\ (t) - I_0$ measured during irradiation at different C_0, where $I_{DS}\ (t)$ is shown in (a) and I_0 is the current before irradiation. (c) Fit of the time evolution of $\Delta I\ (t)$ to the equation $\Delta I\ (t) = \Delta I_{sat} - B \times \exp\left(-\tau_{h\nu,E}\right)$. Black dots are experimental data, red lines are the fitting curves. The curves were fitted starting approximately 7 s after switching on the UV irradiation. During such initial time, a finite MC concentration builds up, triggering the subsequent self-assembly

process (see also Supplementary Discussion 2). The values extracted from the fit for ΔI_{sat}, B and $\tau_{h\nu,E}$ are listed in Supplementary Table 2. We highlight that the minor ΔI recorded for the top left panel in (c) corresponds to a situation in which graphene is covered only by the solvent (1-phenyloctane), see also Fig. S3. Channel size: $L = W = 50$ µm. Bias voltage $V_{DS} = 10$ mV; $V_{GS} = 0$ V.

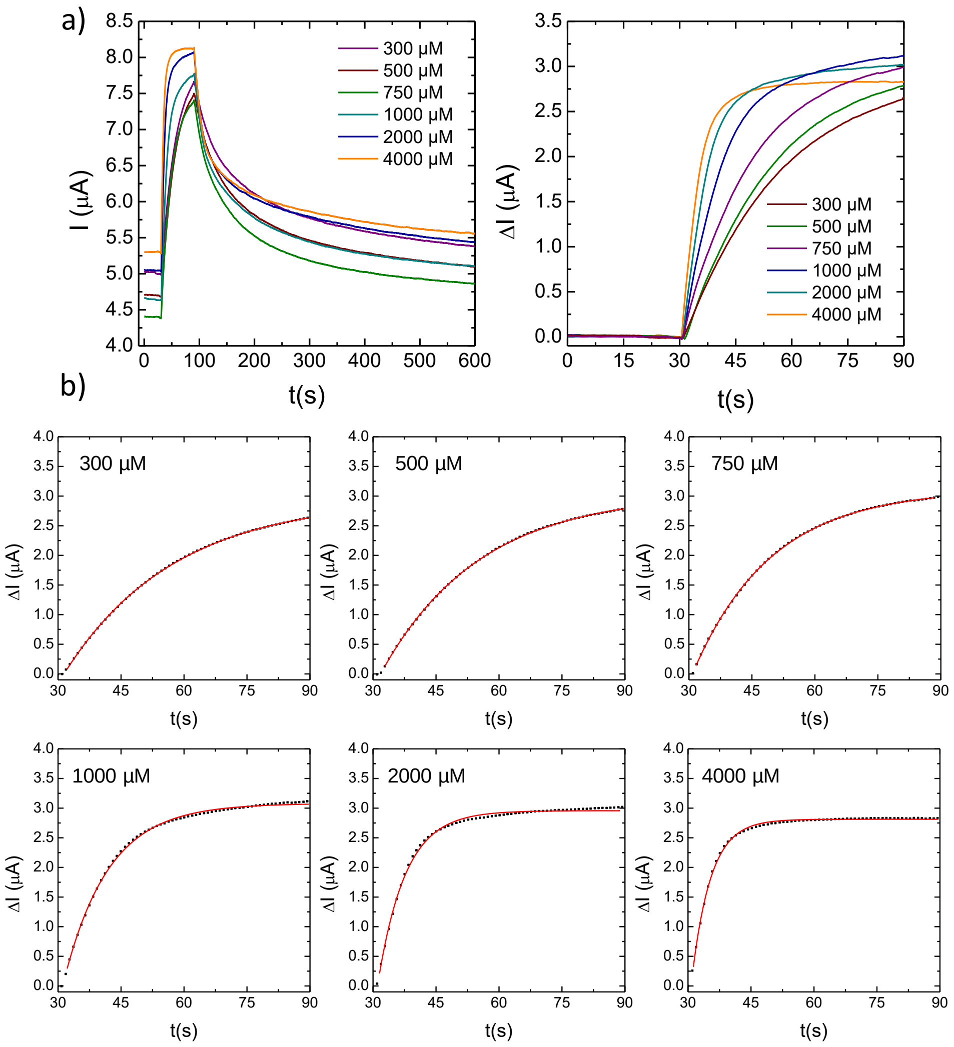

Supplementary Figure 6: Time evolution of the electrical current for a third device at different C_0. (a) Time evolution of current $I_{DS}(t)$ measured during subsequent irradiation/relaxation cycles for another device (hereafter D3) covered by solutions with different SP concentration. For all measurements, UV light was switched on at t = 30 s and off at t = 90 s. (b) ΔI (t) = I_{DS} (t) - I_0 measured during irradiation at different C_0, where I_{DS} (t) is shown in (a) and I_0 is the current before irradiation. (c) Fit of the time evolution of ΔI (t) to the equation ΔI (t) = $\Delta I_{sat} - B \times \exp(-t / \tau_{h\nu,E})$. Black dots are experimental data, red lines are the fitting curves. The curves were fitted starting approximately 7 s after switching on the UV irradiation. During such initial time, a finite MC concentration builds up, triggering the subsequent self-assembly process (see also Supplementary Discussion 2). The values extracted from the fit for ΔI_{sat}, B and $\tau_{h\nu,E}$ are listed in Supplementary Table 2. Channel size: L = W = 100 μm. Bias voltage V_{DS} = 10 mV; V_{GS} = 0 V.

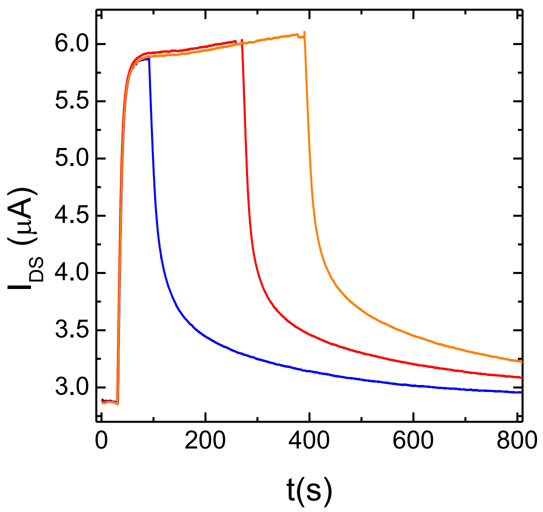

Supplementary Figure 7. Time evolution of the current increase I_{DS} during consecutive irradiation cycles with different irradiation time. Even if the three traces were measured in subsequent irradiation cycles, they are superimposed in Fig. 3e in such a way that the UV light was turned on at the same instant, corresponding arbitrarily to t =30 s. Afterwards, the UV light was switched off in different moment in the different cycles, respectively at t = 90 s, 270 s, 390 s. Importantly, the initial increase in I_{DS} recorded in the three cycles is extremely similar, implying that the dynamics of formation of the self-assembled adlayer are the same in the three cases. Measurements performed at a SP initial concentration C_0 = 4 mM.

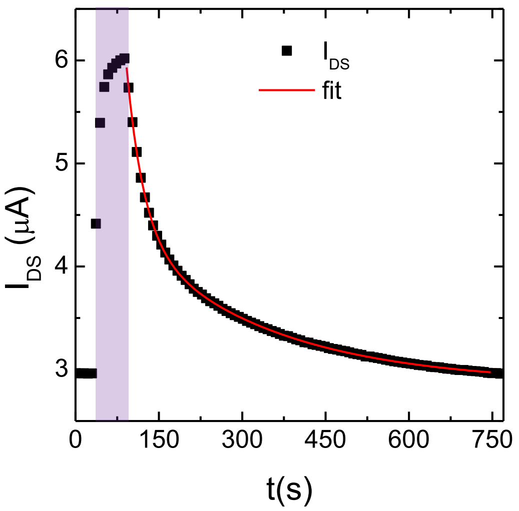

Supplementary Figure 8. Decay of I_{DS} after UV irradiation. The decay of the current after UV irradiation is related to the desorption of the self-assembled adlayer. The decay was fitted by the sum of two exponential decays,

$$I_{DS}(t) = I_0 + A_1 e^{-\frac{t}{\tau_1}} + A_2 e^{-\frac{t}{\tau_2}}$$

In the case shown in Fig. S4, the parameters extracted from the fit were τ_1 = 31 s; τ_2 = 254 s; A_1 = 2.0 μA; A_2 = 31 μA; I_0 = 2.9 μA.

C_0 (μM)	Abs_{sat}	B	$\tau_{h\nu,O}$ (s)
10	0.02	0.02	16.6
25	0.06	0.06	20.4
50	0.13	0.14	22.2
100	0.24	0.26	20.0
150	0.40	0.41	17.2
200	0.51	0.52	19.2
300	0.59	0.62	16.6
500	0.90	0.92	17.2
750	1.12	1.12	19.6
1000	1.39	1.41	18.8
2000	1.9	1.89	18.5
4000	2.1	2.1	17.2

Supplementary Table 1: Parameters extracted from the fit of the photoisomerization kinetics obtained by monitoring the absorbance of SP solutions at different C_0. Parameters extracted by fitting time evolution of absorbance (showed in Fig. S1) to the equation $Abs_{590\ nm}(t) = Abs_{sat} - B \times \exp(-t / \tau_{h\nu,O})$.

	Device D1			***Device D2***			***Device D3***		
**C_0 (μM)**	**ΔI_{sat} (μA)**	**B (μA)**	**$\tau_{h\nu,E}$ (s)**	**ΔI_{sat} (μA)**	**B (μA)**	**$\tau_{h\nu,E}$ (s)**	**ΔI_{sat} (μA)**	**B (μA)**	**$\tau_{h\nu,E}$ (s)**
10	2.8	3.2	256	3.3	3.7	270			
50	2.1	3.1	90	1.9	3.5	52			
100	2.3	4.5	52	2.2	4.8	43			
150	2.5	6.4	35	2.5	3.6	32			
200	2.7	9.0	29	3.1	8.5	29			
300	2.8	9.0	29	3.0	7.8	33	2.9	9.8	26
500	3.2	26	15	3.3	9.0	30	3.0	11	23
750	3.2	53	11	3.4	10	26	3.1	16	18
1000	3.1	100	9.1	3.1	9.6	25	3.1	57	10.6
2000	3.0	140	8.4	3.0	140	8.3	2.9	332	6.5
4000	3.0	679	5.9	3.0	407	6.2	2.8	2570	4.5

Supplementary Table 2: Parameters extracted from the fit of the current flowing through three graphene devices covered by a drop of SP solution with different C_0. The parameters extracted by fitting the time evolution of the current (show in Fig. S3-S5) to the equation $\Delta I (t) = \Delta I_{sat} - B \times \exp(-t / \tau_{h\nu,E})$.

Supplementary Note 1

Synthesis and characterization of the spiropyran derivative[2].

General synthetic methods, materials, and analytical techniques. Ethyl acetate, dichloromethane, petroleum ether and ethanol were distilled before usage. All other starting materials were used as received. NMR spectra were recorded on a Bruker DPX 300 Spectrometer (300 MHz for ^1H, 75 MHz for ^{13}C) at 25 °C using residual protonated solvent signals as internal standard (^1H: δ($CDCl_3$) = 7.26 ppm; δ(DMSO-d_6) = 2.50 ppm. The splitting patterns are abbreviated as follows: singlet (s), doublet (d), triplet (t), quadruplet (q), multiplet (m), and broad (br). UPLC/MS was performed with a Waters UPLC Acquity equipped with a Waters LCT Premier XE Mass Detector for UPLC-HR-MS, with Waters Alliance systems (consisting of a Waters Separations Module 2695, a Waters Diode Array Detector 996 and a Waters Mass Detector ZQ 2000). Column chromatography was carried out with silica gel (Merck 60, particle size 0.040-0.063 mm) using eluents as specified.

*Synthetic procedures. Synthesis of 1',3'-Dihydro-1'-octadecyl-3',3'-trimethyl-6-nitrospiro[2H-1-benzopyran-2,2'-(2H)-75 indole]*This compound was synthesized by adapting a literature procedure.[1] 2,3,3-trimethyl-3H-indole (2.0 mL, 12.6 mmol) was treated with 1-iodooctadecane (5.5 g, 13.8 mmol) in acetonitrile (12 mL) to give a tan suspension. While heating to 80 °C the reaction went into solution. After five days the reaction was cooled to room temperature and appeared as suspension. Acetonitrile was removed under reduced pressure and the remaining solid was washed intensively with EtOAc under sonication. This residue was dissolved in dichloromethane and washed with 1 M aqueous NaOH solution. The organic phase was dried over anhydrous $MgSO_4$ and evaporated under reduced pressure. The resulting crude product was purified by column chromatography (silica, PE/EtOAc = 96:4) to yield the product (5.1 g, 9.5 mmol, 76%) as a white solid. ^1H NMR (300 MHz, DMSO-d6): δ 7.97 (m, 1H), 7.85 (m, 1H), 7.62 (m, 2H), 4.44 (t, 2H), 1.81 (m, 2H), 1.53 (s, 6H), 1.22 (m, 32H), 0.84 (t, 3H); HRMS (m/z): $[M]^+$ calcd. for $C_{28}H_{49}N$, 412.3938; found, 412.3812.

*Synthesis of 1',3'-Dihydro-1'-octadecyl-3',3'-trimethyl-6-nitrospiro[2H-1-benzopyran-2,2'-(2H)-indole].*This compound was synthesized by adapting a literature procedure[2]. 1-Octadecyl-2-methylene-3,3-dimethylindoline (2.6 g, 6.3 mmol) was treated with 2-hydroxy-5-nitrobenzaldehyde (1.2 g, 6.9 mmol) in 25 mL of EtOH to give a tan suspension. While heating the reaction to 80 °C overnight the suspension went into solution. After cooling to room temperature, the mixture was diluted with water and EtOAc and separated. The aqueous phase was back extracted with EtOAc. Combined the organic layers and washed with water and saturated aqueous NaCl solution. The organic phase was dried over anhydrous $MgSO_4$ and evaporated under reduced pressure. The resulting crude product was purified by column

chromatography (silica, PE/EtOAc 9:1) to yield the product (1.73 g, 3.1 mmol, 50%) as a white solid. Characterization data agree with the literature[2]. ^{1}H NMR (300 MHz, $CDCl_3$): δ 8.03 (d, J = 2.7 Hz, 1H), 8.00 (m, 1H), 7.19 (dt, J = 7.5 Hz, 1.2 Hz, 1H), 7.09 (dd, J = 7.2 Hz, 1.2 Hz, 1H), 6.90 (d, J = 10.2 Hz, 1H), 6.87 (dt, J = 7.2 Hz, 0.9 Hz, 1H), 6.74 (d, J = 8.7 Hz, 1H), 6.57 (d, J = 7.5 Hz, 1H), 5.86 (d, J = 10.5 Hz, 1H), 3.14 (m, 2H), 1.26 (m, 32H), 1.19 (s, 3H), 0.89 (t, J = 6.9 Hz, 3H); HRMS (m/z): $[M]^+$ calcd. for $C_{36}H_{52}N_2O_3$, 561.4051; found, 561.4059.

Supplementary Discussion 1

Origin of the molecular induced gating effect

We previously reported that a MC adlayer at the solid-air interface introduced n-type doping on graphene, which was understood on the basis of the nanoscale molecular arrangement.[2] In particular, molecular dynamics simulations showed that MC isomers in the assembly are slightly tilted with respect to the basal plane of the substrate. Such tilt gives rise to a non-zero out-of-plane component of each molecular electrical dipoles, which sum up to generate an electric field effect capable of shifting the work function of graphene, as a gate terminal. Since the nanoscale assembly of MC is the same at the solid/liquid and at the solid/air interface, a similar effect is measured in this work. We also stress that the ordered structure framed by self-assembly ensures that each molecule contributes with the same nanoscale field to the overall molecular gating effect. Therefore, the intensity of the overall field effect will be proportional to the number of molecules on the graphene surface (see next section), that is, ΔI scales linearly with the covered area. The same field-effect on graphene is induced by different crystalline domains of the molecular adlayer with lamellae oriented along the equivalent crystallographic directions of graphene. Indeed, the gating effect originates from the out-of-plane component of the electrical dipoles, which does not depend on the in-plane orientation of the lamellar assembly. In this regard, we consider that the maximum shift in charge neutrality point is achieved when the graphene layer is fully covered – so that the field effect acts all over the graphene surface. If instead part of the graphene surface is left uncovered, it maintains its original charge neutrality point and it does not contribute to the ΔI increase.

We highlight that a linear change of the electrical current with the surface coverage is not a specificity of our system. Rather, the same scaling is expected for every situation in which a self-assembled adlayer introduces a field effect in graphene *via* ordered alignment of permanent dipoles. Additionally, a very similar situation is expected for all molecular processes which generate a variation of ordered (out-of-plane) dipoles at the graphene surface. In this regard, we anticipate that our method and our machinery for data analysis might allow monitoring the

dynamics not only of other self-assembled adlayers, but also of other interfacial phenomena, such as 2D polymerization.

On the contrary, the absence of an ordered assembly for the SP isomer at the solid/liquid interface implies that molecular dipoles in the vicinity of the surface are randomly oriented at any moment in time, hence they do not introduce a strong field effect. We highlight that at the solid/air interface we were able to visualize a self-assembled adlayer for the same SP derivative, in which alkyl chains were imaged with high resolution, while the SP head groups gave rise to fuzzy regions. Our interpretation of those images, supported by molecular dynamics simulations, was that SP isomer were not immobilized on the surface, even though the alkyl chains anchored them on the substrate. In the present case, the situation at the solid/liquid interface is more dynamic, as molecules are more mobile; hence, we are not capable of imaging even the alkyl chains.

Supplementary Discussion 2

Model for the dynamics of self-assembly

The increase ΔI in current flowing through graphene can be understood as the result of a gating effect induced by MC molecules. In particular, considering that (i) all MC molecules introduce the same electric field effect on graphene and (ii) the I_{DS}-V_G trace of graphene is linear at V_G = 0 V (see methods section), we can consider ΔI proportional to the number of MC molecules on the graphene surface N_{sub}:

$$\Delta I \sim N_{sub} \tag{1}$$

In order to describe the variation of N_{sub}, during the formation and desorption of the self-assembled adlayer, we employ a modified version of a previously developed model[3,4] and describe the variation in N_{sub} on the basis of molecular adsorption and desorption.

The adsorption is proportional to the number of MC molecules in solution N_{sol}, the adsorption rate K_{ads} and the number of available spots on the substrate surface. In turn, the number of available spots on the surface can be described as the difference between the maximum number of molecules which fit on the surface and the molecules on the surface at a given moment in time N. The maximum number of molecules which fit on the surface is given by the total substrate area A_{sub} divided by the area occupied by one molecule A_{mol}.

The desorption is proportional to the number of MC molecules on the substrate N_{sub} times the desorption rate K_{des}. Thus, one can write:

$$\frac{dN_{sub}(t)}{dt} = K_{Ads}\left(\frac{A_{sub}}{A_{mol}} - N_{sub}(t)\right)N_{sol}(t) - K_{Des}N_{sub}(t) \tag{2}$$

In which the first term describes the adsorption of MC molecules from solution, and the second term the desorption of the MC molecules from the substrate. It is worth noting that during photoisomerization, the number of MC molecules N_{sol} is not constant, but it varies with time. In particular, during UV irradiation the MC concentration in solution increases, following the exponential trend which can be monitored by the absorbance:

$$N_{sol}(t) = N_{sta}\left(1 - e^{-\frac{t}{\tau_{h\nu,O}}}\right) \tag{3}$$

Where N_{sta} is the number of MC molecules at the stationary state, reached when the SP → MC photoisomerization is balanced by the MC → SP thermal conversion, and $\tau_{hv,O}$ is the time constant extracted from the optical characterization, as defined in the main text. Instead, after UV irradiation, the number of MC molecules in solution decays as:

$$N_{sol}(t) = N_{sta}\, e^{-\frac{t}{\tau_{\Delta,O}}} \quad (4)$$

Where $\tau_{\Delta,O}$ is the time constant extracted from the optical characterization for the thermal decay. Substituting the concentration dependence into (1), one obtains a non-homogeneous first order differential equation which does not have any trivial exponential solution.

However, one can understand the exponential dependence of the current during UV irradiation by considering, as a rather brute approximation, that the number of MC molecules does not varies with time, but it is rather constant at a value $N_{sol} \sim C$. In this case, one can rewrite Eq. (2) as:

$$\frac{dN_{sub}(t)}{dt} = K_{Ads}C\,\frac{A_{sub}}{A_{mol}} - (K_{Ads}C + K_{Des})N_{sub}(t) \quad (5)$$

which has solutions in the form:

$$N_{sub}(t) \sim const - e^{-(K_{Ads}C + K_{Des})t} \quad (6)$$

High initial SP concentration C_0 will generate a high number of MC C, so that C will be higher for higher C_0, in agreement with the electrical measurements. We point out that the approximation of constant $N_{\mathrm{sol}} \sim C$ fails immediately after switching on the UV light. Indeed, at the very beginning of the irradiation, the MC concentration is zero, and it requires a certain time before reaching a finite concentration. During such time, the relative variation in MC concentration is maximal. Instead, after a relatively short time (a few s) a critical MC concentration is reached, and further increase in N_{sol} become less relevant. In this regard, by fitting the $I_{\mathrm{DS}}(t)$ curves after the initial time required for a significant MC concentration to build up (see Figs. S4-S6), we capture the dynamics of assembly formation with the simple exponential fit.

In the light of this discussion, another interesting observation can be extracted by comparing the decay in $I(t)$ related to the desorption of the self-assembled MC adlayer and the decay in absorbance, as reported in Fig. 3e. During approximately one minute after switching off the UV light, the decay in $I(t)$ is faster than that in Abs(t). On the contrary, according to equation 1, one

would expect that the desorption of the self-assembled adlayer were slower than the thermal recovery in solution – which would reflect a physical situation in which desorption takes place after the density of MC in solution has decreased significantly. This indicates that there is a physical phenomenon which favors the desorption of the self-assembled adlayer at a timescale faster than the thermal reconversion in solution, which is not contained in equation (2). While our experiment does not provide direct information on such phenomenon, we postulate that it is the MC $\rightarrow$ SP conversion occurring on the surface rather than in the solution, which leads to the dissolution of the MC assembly following other (faster) dynamics.

Supplementary Discussion 3

Ensemble dynamics

Ensemble molecular processes involving a large number of molecules, such as photoisomerization in solution or the formation of self-assembly, typically evolve according to relatively slow time constants, ranging from the ms to the s. While (ultra)fast molecular events take place at the single molecule level, the dynamics of systems composed of a large number of molecules are typically dominated by ensemble quantities describing population-averaged stochastic processes[5], which lead to orders-of-magnitude slower dynamics. For example, while at the single molecule level light-induced isomerization of photochromes takes place at an ultra-fast timescale (fs-ns)[6,7], at the ensemble level the dynamics are determined by absorption and photoisomerization quantum yield[8,9], leading to ensemble dynamics in the few-seconds range[10]. Analogously, the phenomena governing the motion of single molecules on surfaces (vibrations and hopping rates) occur at the ps timescale at room temperature,[11] while self-organized supramolecular assemblies composed of a large number of molecules evolve on slower (few-seconds) timescales, which are ideal to be captured by our device method using a 10 - 100 Hz sampling rate easily achievable with conventional electronics.

Supplementary References

1. Luo, Z., Pinto, N. J., Davila, Y. & Charlie Johnson, a. T. Controlled doping of graphene using ultraviolet irradiation. *Appl. Phys. Lett.* **100**, 253108 (2012).
2. Gobbi, M. *et al.* Collective molecular switching in hybrid superlattices for light-modulated two-dimensional electronics. *Nat. Commun.* **9**, 2661 (2018).
3. Yokoyama, S., Hirose, T. & Matsuda, K. Phototriggered formation and disappearance of surface-confined self-assembly composed of photochromic 2-thienyl-type diarylethene: a cooperative model at the liquid/solid interface. *Chem. Commun.* **50**, 5964–5966 (2014).
4. Yokoyama, S., Hirose, T. & Matsuda, K. Effects of Alkyl Chain Length and Hydrogen Bonds on the Cooperative Self-Assembly of 2-Thienyl-Type Diarylethenes at a Liquid/Highly Oriented Pyrolytic Graphite (HOPG) Interface. *Chem. – A Eur. J.* **21**, 13569–13576 (2015).
5. McQuarrie, D. A. *Statistical mechanics*. (University Science Books, 2000).
6. Waldeck, D. H. Photoisomerization dynamics of stilbenes. *Chem. Rev.* **91**, 415–436 (1991).
7. Polli, D. *et al.* Conical intersection dynamics of the primary photoisomerization event in vision. *Nature* **467**, 440 (2010).
8. Keitaro, N., Jonathan, P., Pei, Y. & Rémi, M. Introduction: Organic Photochromic Molecules. in *Photochromic Materials* 1–45 (Wiley-Blackwell, 2016). doi:10.1002/9783527683734.ch1.
9. Stranius, K. & Börjesson, K. Determining the Photoisomerization Quantum Yield of Photoswitchable Molecules in Solution and in the Solid State. *Sci. Rep.* **7**, 41145 (2017).
10. Klajn, R. Spiropyran-based dynamic materials. *Chem. Soc. Rev.* **43**, 148–184 (2014).
11. Barth, J. V. Transport of adsorbates at metal surfaces: from thermal migration to hot precursors. *Surf. Sci. Rep.* **40**, 75–149 (2000).
